# Using Network Methodology to Infer Population Substructure

**DOI:** 10.1371/journal.pone.0130708

**Published:** 2015-06-22

**Authors:** Dmitry Prokopenko, Julian Hecker, Edwin Silverman, Markus M. Nöthen, Matthias Schmid, Christoph Lange, Heide Loehlein Fier

**Affiliations:** 1 Institute of Genomic Mathematics, University of Bonn, Bonn, Germany; 2 Department of Biostatistics, Harvard School of Public Health, Boston, United States of America; 3 Channing Laboratory, Brigham and Women's Hospital, Boston, United States of America; 4 German Center for Neurodegenerative Diseases (DZNE), Bonn, Germany; 5 Institute of Human Genetics, University of Bonn, Bonn, Germany; 6 Institute of Medical Biometrics, Informatics and Epidemiology, University of Bonn, Bonn, Germany; Case Western Reserve University, UNITED STATES

## Abstract

One of the main caveats of association studies is the possible affection by bias due to population stratification. Existing methods rely on model-based approaches like *structure * and ADMIXTURE or on principal component analysis like EIGENSTRAT. Here we provide a novel visualization technique and describe the problem of population substructure from a graph-theoretical point of view. We group the sequenced individuals into triads, which depict the relational structure, on the basis of a predefined pairwise similarity measure. We then merge the triads into a network and apply community detection algorithms in order to identify homogeneous subgroups or communities, which can further be incorporated as covariates into logistic regression. We apply our method to populations from different continents in the 1000 Genomes Project and evaluate the type 1 error based on the empirical p-values. The application to 1000 Genomes data suggests that the network approach provides a very fine resolution of the underlying ancestral population structure. Besides we show in simulations, that in the presence of discrete population structures, our developed approach maintains the type 1 error more precisely than existing approaches.

## Introduction

Within the last decade, genome wide association studies (GWAS) have shown to be a powerful analytical tool in association mapping to identify common variants (i.e. variants with minor allele frequencies of more than 1%) that contribute to the heritability of complex diseases[1]. The GWAS methodology thereby utilizes the underlying, population specific linkage-disequilibrium structure of common variants and thus only a limited number of variants needs to be genotyped in order to capture the common variation within a population[2,3].

Although a large number of GWAS for various complex diseases have been conducted up to date, for most complex diseases, the proportion of the estimated variance that can be explained by common variation is rather low [4–6]. Besides alternative factors that are likely to contribute to the estimated heritability of a trait like gene-gene interactions, variants with low frequencies, environmental factors, etc., it has also been suggested that so far undetected common variants with low penetration rates could further increase the proportion of estimated genetic variance for complex diseases, although it is widely agreed that for most complex diseases, those low-penetration rate common variants that have not been detected so far are likely to show smaller effect sizes compared to the known common risk variants[3,7,8].

Park et al. [9] show on the basis of reported GWAS findings for height, Crohn’s disease, and breast, prostate and colorectal (BPC) cancers, that there are likely to be additional common variants with low penetration rates and rather small effect sizes that could still explain more than 15–20% of the estimated heritability of the traits. In order to detect those common variants with low penetration rates however, large sample sizes for GWAS are required to ensure sufficient power. Technological advances in genotyping technologies along with a rapid cost decline for genotyping services and the establishment of large research consortia have paved the way for those large-scale GWAS. Some recent examples of GWAS with large sample sizes that have identified new susceptibility loci in common variation include association studies on breast cancer risk [10], coronary artery disease [11,12], or ovarian cancer [13].

In most of the cases, those large-scale GWAS have a population-based instead of a family-based design, since the population based design has a greater power to detect causative loci[14] and the sample collection for population based designs is much easier, especially for late on set diseases [15]. One pitfall of population-based association studies in contrast to family-based association studies is however, that they bear the risk of population stratification. Population stratification refers to a non-homogeneous distribution of alleles among subjects in the sample due to ancestral differences and can bias the true association structure between the trait and the sampled genotypes[16]. It is thereby likely that the risk of population stratification for a genetic association study increases with its sample size, since the larger the sample size, the more difficult it becomes to ensure a homogenous ancestral background of the sampled individuals [17]. Even in a relatively homogenous study design the issue of population structure might affect the results [18,19].

There exist several methodological approaches on how to correct for population stratification in genetic association studies. One is the Genomic Control [20], which is based on the chi-square scores of the association tests. Genomic Control shows how inflated the calculated scores are, compared to the null distribution. It thereby assumes a constant inflation rate over the tested region, which might not always be the case, especially for large genomic regions. Next, there exist a group of model-based approaches, which estimate the population structure based on the observed genotypes. Two examples of those model-based approaches include STRUCTURE [21] and ADMIXTURE [22]. Both methods allow for fractional subpopulation memberships, but ADMIXTURE is more efficient in terms of runtime. Finally, another stream in the literature suggests to first calculate the genetic covariance matrix between studied individuals and then extract the principal components, which are further used as continuous covariates in association analysis [23–25]. There are several extensions of the PCA approach. Some of them are justified by the fact that using only continuous covariates might be not enough[26–28]. They try to extend the method by using also discrete membership covariates, obtained from clustering approaches. Other methods include mixed models with fixed and random effects to model the outcome variable[29,30]. Here the population structure is modeled as a random effect. It is important to mention that combining mixed models with principal component covariates can increase the quality of correction for population structure[31,32].

Rosenberg and Nordborg [33] showed that the occurrence of false positive associations is a serious problem in mixtures of discrete subpopulations, whereas in an admixed population it depends on the variance of admixture between individuals. When the variance is small, spurious associations are less severe.

In this manuscript we use network methodology to infer and to visualize population structure in genotype data. Normally, visual inspection of GWAS data is performed by creating a PCA or MDS plot. Here, we present a different way to visualize the ancestral population structure and possible batch artifacts. The intuition behind the method is well suited for a dataset, consisting of several discrete subpopulations, hence in this setting the method performs particularly well. The identified subpopulations can be subsequently included as discrete covariates into association analysis. We apply our method to different populations in the 1000 Genomes Project and evaluate our type 1 error compared to standard methodology in simulation studies. All calculations and simulations are implemented in R with the help of igraph package and Eigensoft[34,24].

## Methods

Here we present an algorithm, how to build a social graph between individuals in a genotypic dataset. The main idea of the method is based on social network methodology. In order to identify closely related individuals in a given population, we group them into triads based on a predefined similarity measure, and then combine those triads into a graph for further analysis.

In terms of social network theory, triads can be thought of as the smallest social community, they consist of three individuals and describe the pairwise relations among themselves [35]. Following the existing theory on triads, those three individuals can be unconnected (empty triad), can be partially connected (one-edge triad, two-path triad), or all three individuals can be connected to each other (triangle triad).Since we aim to identify individuals that are closely linked to each other on the basis of their genetic profile, we utilize the triangle-structure of transitive triads to create the triplets, and thus define that for three individuals (A, B, C), not only A and B, and B and C, but also A and C have to be linked to each other based on their genetic profile.

We will further outline how we derive the social graph for a given set of individuals based on their genetic profile, and then describe how this graph can be partitioned into communities which can be utilized as covariates in association analysis.

Given n individuals and m biallelic markers we display them in a genotype matrix GT:

$$GT=\left(\begin{array}{ccc} g_{11} & \ldots & g_{1m}\\ \vdots & \ddots & \vdots \\ g_{n1} & \ldots & g_{nm} \end{array}\right),g_{i,k}\in \{0,1,2\}$$ (1)

For the triad construction we need a similarity measure between individuals in order to identify their degree of "relatedness". This can be a simple Euclidian distance between genotype vectors [36] or a variance-covariance matrix between individuals [23,24]. For the sake of simplicity, we further outline our approach on the basis of the variance-covariance matrix, although it is important to note, that in principle any other similarity or distance matrix could be used.

Let D represent the genetic covariance matrix, calculated as described in Price et al. [24]. It is an nxn symmetric matrix and D_ij_ is the covariance between individuals i and j.

A triad in terms of graph theory can be defined as an undirected graph G = (V,E), where V is the vertex set (set of individuals) and E is the edge set (structure of connections):

G=(V,E),V={1,2,3},E={{1,2},{2,3},{1,3}} (2)

For every individual i, we aim to find two other individuals j and k, so that the covariances between the three individuals are maximized in a stepwise manner. In order to simplify this pairwise optimization problem, we rank the other individuals based on their pairwise covariance with individual i as shown in Table 1. Those individuals that show the highest covariance with individual i are assigned the highest ranks, and those that show the smallest covariance are assigned the lowest ranks.

**Table 1:** Ranking procedure for individual i.

Subject	Covariance value with i	Rank
1	0.004918	4.5
2	0.014093	2
3	0.000124	6
4	0.028862	1
5	0.004918	4.5
6	0.012716	3
. . .	. . .	. . .

### Step 1

We first want to find the closest subject j to i. In order to do this we minimize the term (3).

$$\underset{j}{\text{min}}(rank^{\left(i\right)}[j]+rank^{\left(j\right)}[i])$$ (3)

Here *rank* ^(*i*)^[*j*] represents the relative position of subject j for subject i and *rank* ^(*j*)^[*i*] represents the relative position of subject i for subject j based on the covariance matrix D. Such "two-sided" optimization allows us to account for two-sided "closeness" from i to j and from j to i. This gives us the advantage to create more robust pairs in terms of relatedness.

### Step 2

We now want to find the third subject k in the triad. This subject should be closely related to both already included individuals i and j. In order to fulfill this requirement we again use the term (3), but for both dyads: {i,k} and {j,k}:

$$\underset{k}{\text{min}}(rank^{i}[k]+rank^{k}[i]+rank^{j}[k]+rank^{k}[j])$$ (4)

In such a way we make sure that the individual k is similar to both individuals in the dyad {i,j}.

After creating triadic groups for all n subjects, we merge the retrieved triads in a graph that will contain n vertices and 3*n edges and that can be further partitioned into communities by using community-detection algorithms.

### Community detection

After a first look on the created graph, one can already determine robust groups by identifying unconnected components of the graph. Those components, as we show in our simulation studies, already present a valid partition into groups, especially with data, coming from discrete populations. In order to increase the resolution of clustering into groups within those unconnected components we further outline how communities within the network can be detected.

The community structure of a network or graph can be seen as groups of nodes which are densely connected within their community and sparsely connected between groups[37]. The simplest community detection analysis on a graph would be identifying its unconnected components. More sophisticated community-detection algorithms aim to measure the modularity, which shows the quality of a particular division of the network in communities [37]. It is defined as:

$$Q=\sum_{i=1}^{k}\left(e_{ii}-a_{i}^{2}\right)\;,$$ (5)

where *e* is a kxk matrix, whose entries *e*_*ij*_ represent the fraction of connections between communities i and j, and $a_{i}=\sum_{j=1}^{k}e_{ij}$, k is the number of communities.

The modularity is calculated in such a way that it compares the fraction of connections within communities in a graph with the expected value of this fraction, given random connections, but the same community division. A value of 1 would represent a perfect community grouping.

We use the Louvain algorithm from Blondel et al. [38] which is computationally fast and doesn't require a predefined number of communities to be specified in advance. Initially, each node is assigned to its own community. Then, at each step a node is replaced to a neighboring community by maximizing the modularity gain with this replacement. The process stops if there is no further gain possible or there is only one community left. For typical and sparse data the computational time is linear and depends on the number of nodes. Blondel et al. applied the algorithm to a network with 118 million nodes and the computational time was 152 minutes. In case of genotype data the computational time of the community detection algorithm breaks down to milliseconds. For example for the European dataset of 378 individuals it took 0.02 seconds to calculate the communities, given a precalculated covariance matrix. The computational complexity is driven by calculation of the covariance matrix between the genotypes of individuals and is quadratic on the number of individuals.

## Results

### Data description and application

We applied our method to the phase 1 data of the 1000 Genomes Project [39]. The 1000 Genomes project offers a unique platform for sequence data analysis since the project provides data for more than 1000 sequenced genomes from various populations. We divided the whole dataset into 4 subsamples, according to the continental membership: Europeans, Africans, Americans, Asians. We applied minor allele frequency filtering with a threshold of 5% and a linkage disequilibrium pruning step. During this step we also excluded long-range LD regions [40]. After data cleaning we were left with 4 datasets, described in Table 2. We then applied our method to every dataset in order to detect subpopulations.

**Table 2:** Description of datasets, used in the analysis.

Dataset	Number of individuals	Number of subpopulations	Number of SNPs (MAF>0.05)
1) Americans	174	3	930369
2) Africans	229	3	2011030
3) Asians	279	3	716976
4) Europeans	378	5	851672

**Fig 1:**
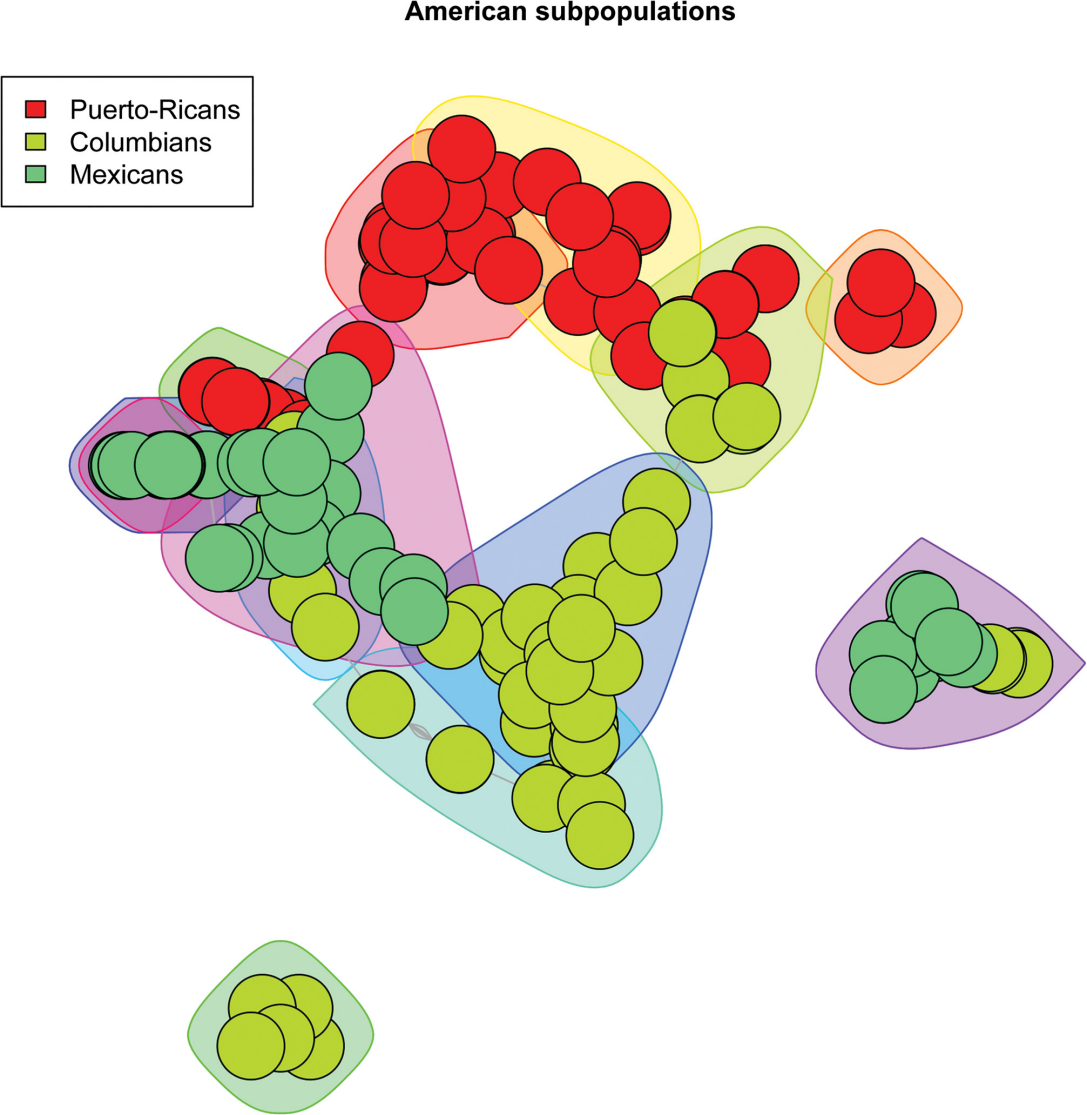
3 American subpopulations. The polygons around the nodes represent the detected communities. The node colors represent the actual labels.

**Fig 2:**
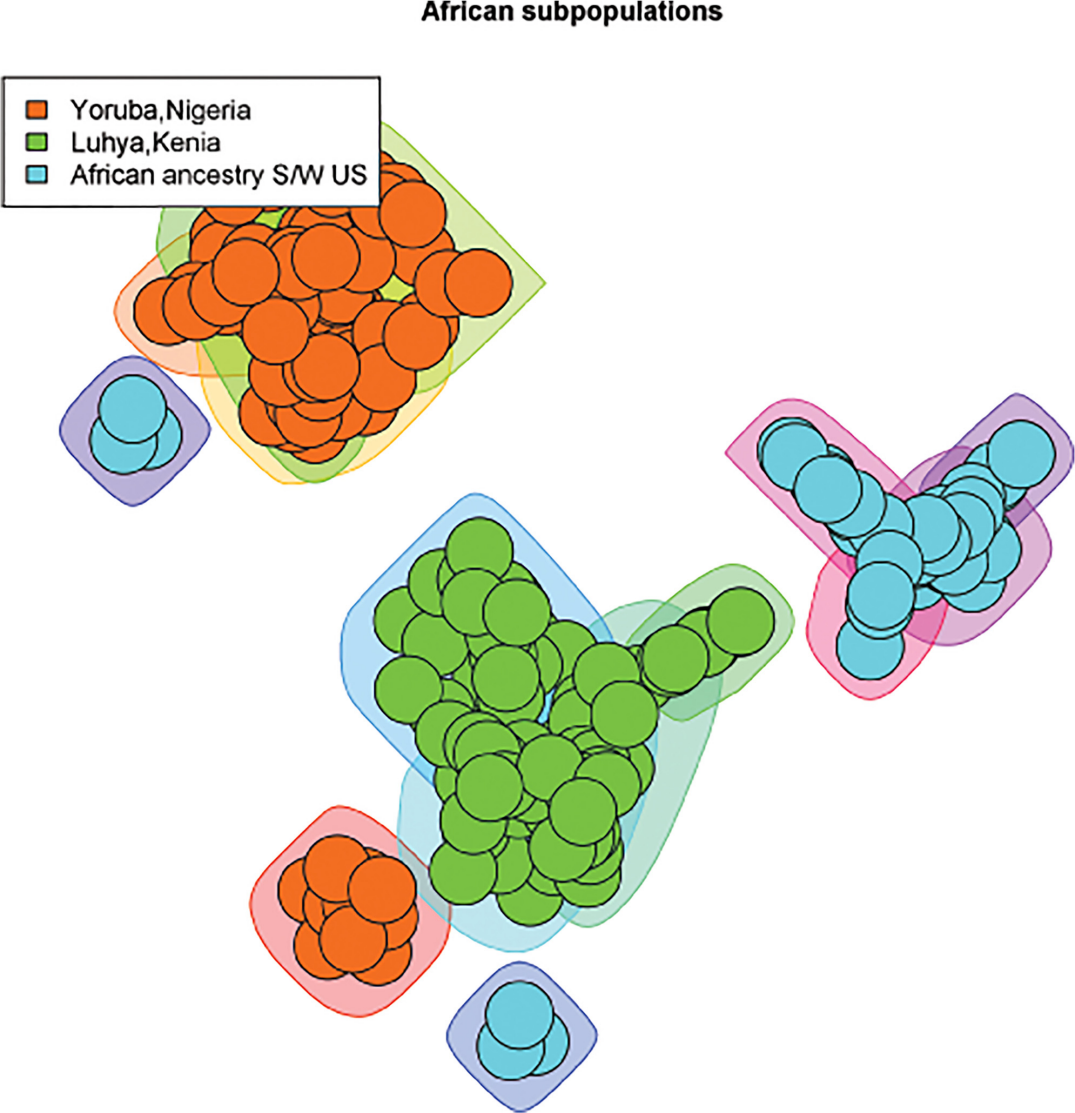
3 African subpopulations. The polygons around the nodes represent the detected communities. The node colors represent the actual labels.

**Fig 3:**
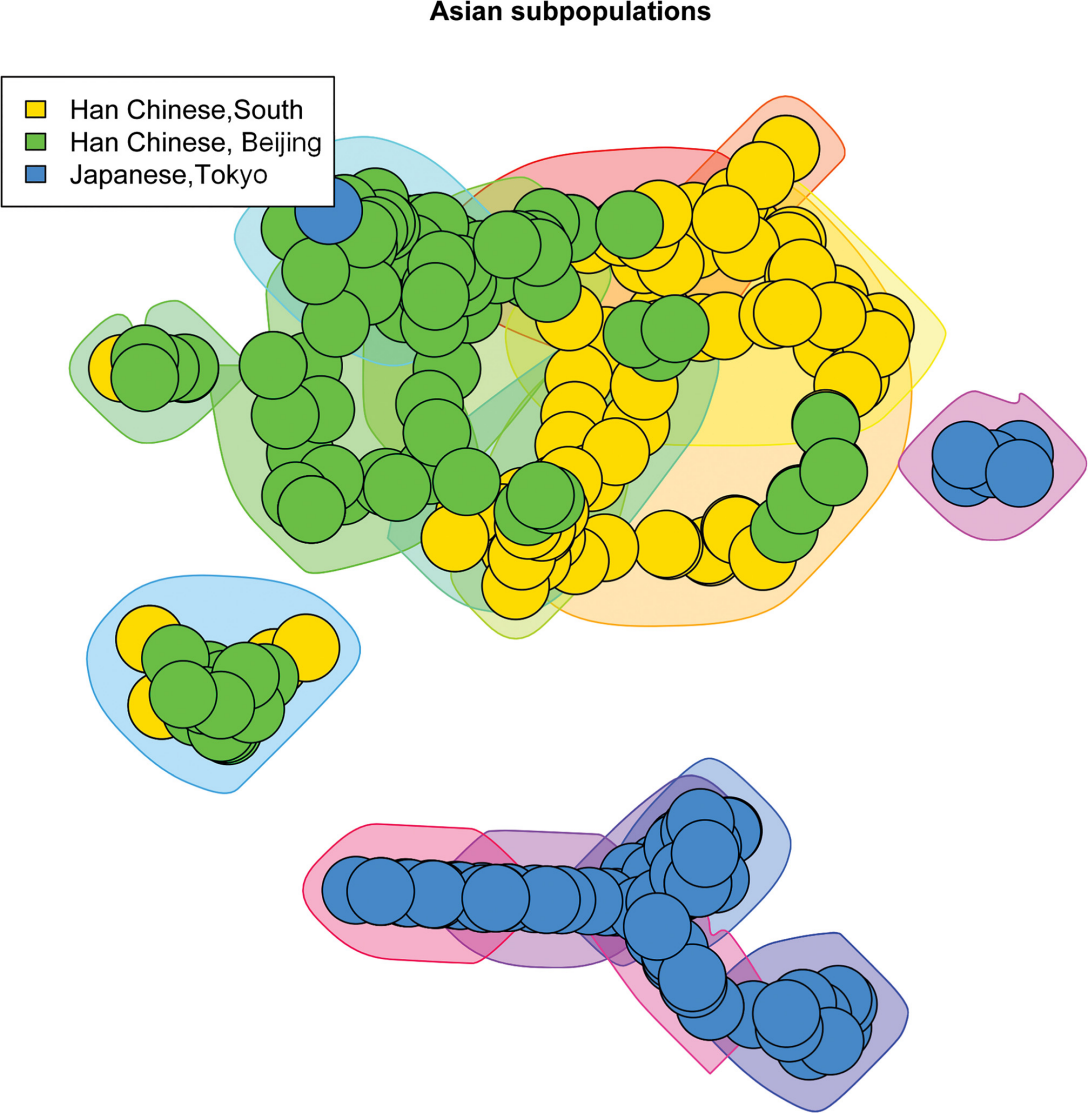
3 Asian subpopulations. The polygons around the nodes represent the detected communities. The node colors represent the actual labels.

**Fig 4:**
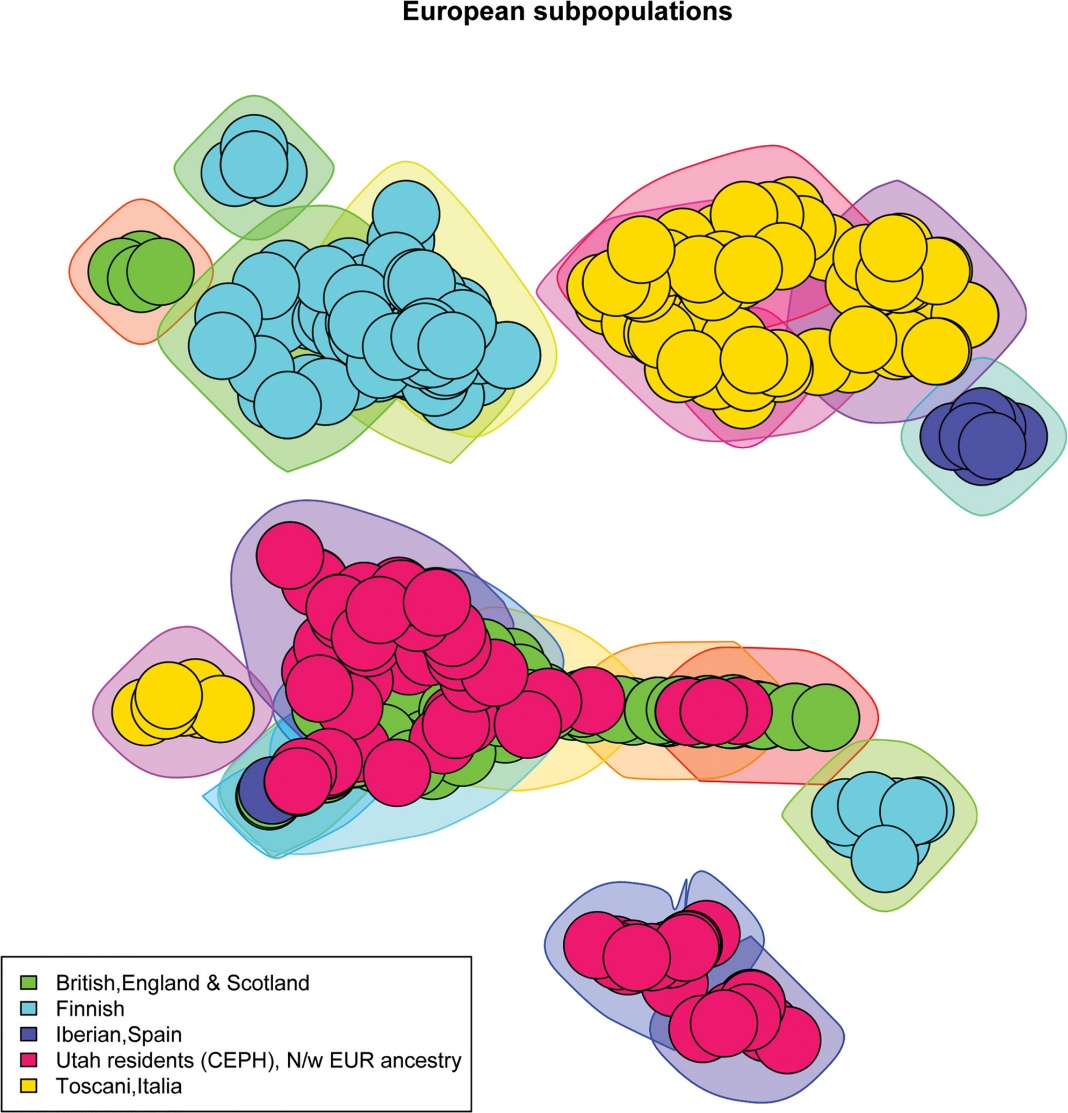
5 European subpopulations. The polygons around the nodes represent the detected communities. The node colors represent the actual labels.

We created scale-free plots of the constructed graphs in Figs 1–4. Every node represents an individual and the color of the node is related to the actual population label of this individual. For purposes of visualization we do not show the edges in the plots. In order to visualize the communities we constructed polygons around the nodes, which are assigned to this community. Every polygon represents 1 community. Full community assignment and the precision we described in Tables 3–6. One can clearly see an almost perfect separation of subpopulations in Africans and Americans. The separation in Asian subpopulations is slightly worse, there are some admixed communities, consisting of Han Chinese from Beijing and from the South. In the European subpopulations we see that the Finnish and Toscanian communities are very homogeneous. The 5 small heterogeneous communities mostly contain individuals from Utah and Great Britain, but this is expected,because the Utah residents from this dataset are known to have a high degree of shared ancestry with the British. For completeness we also included S1–S4 Tables which represent precision for unconnected components of the graphs. One can already determine a good separation for the subpopulations by looking only at the unconnected components.

**Table 3:** Contingency table for American subpopulations, rows correspond to detected communities, columns to actual subpopulations.

	CLM	MXL	PUR
**1**	0	0	18
**2**	0	0	3
**3**	0	0	10
**4**	7	0	8
**5**	0	0	15
**6**	5	0	0
**7**	10	0	0
**8**	10	0	0
**9**	23	0	0
**10**	0	30	0
**11**	5	10	0
**12**	0	16	1
**13**	0	3	0

PUR—Puerto Rican, CLM—Colombian, MXL–Mexican

**Table 4:** Contingency table for African subpopulations, rows correspond to detected communities, columns to actual subpopulations.

	ASW	LWK	YRI
**1**	0	0	12
**2**	0	0	11
**3**	0	0	19
**4**	0	0	16
**5**	0	0	14
**6**	0	0	15
**7**	0	7	0
**8**	0	19	0
**9**	0	27	0
**10**	0	34	0
**11**	3	0	0
**12**	3	0	0
**13**	5	0	0
**14**	25	0	0
**15**	10	0	0
**16**	9	0	0

YRI—Yoruba in Nigeria, LWK—Luhya in Kenia, ASW—African ancestry in Southwest US

**Table 5:** Contingency table for Asian subpopulations, rows correspond to detected communities, columns to actual subpopulations.

	CHB	CHS	JPT
**1**	6	11	0
**2**	0	5	0
**3**	4	20	0
**4**	2	21	0
**5**	0	14	0
**6**	17	0	0
**7**	18	0	0
**8**	4	2	0
**9**	5	12	0
**10**	25	0	1
**11**	16	8	0
**12**	0	0	17
**13**	0	0	11
**14**	0	0	13
**15**	0	0	14
**16**	0	0	5
**17**	0	0	13
**18**	0	0	15

CHS—Southern Han Chinese, CHB—Han Chinese in Beijing, JPT—Japanese in Tokyo

**Table 6:** Contingency table for European subpopulations, rows correspond to detected communities, columns to actual subpopulations.

	CEU	FIN	GBR	IBS	TSI
**1**	0	0	14	0	0
**2**	0	0	4	0	0
**3**	8	0	16	0	0
**4**	5	0	22	0	0
**5**	0	32	0	0	0
**6**	0	26	0	0	0
**7**	0	13	0	0	0
**8**	0	16	0	0	0
**9**	0	5	0	0	0
**10**	0	0	0	6	0
**11**	0	0	0	8	0
**12**	8	0	14	0	0
**13**	3	1	9	0	0
**14**	6	0	8	0	0
**15**	17	0	0	0	0
**16**	12	0	0	0	0
**17**	26	0	1	0	0
**18**	0	0	0	0	24
**19**	0	0	0	0	9
**20**	0	0	0	0	47
**21**	0	0	0	0	4
**22**	0	0	0	0	14

GBR—British in England and Scotland, FIN—Finnish, IBS—Iberian in Spain, CEU—Utah residents with Northern and Western European ancestry, TSI—Toscani in Italy

### Evaluation via simulated association studies

In order to evaluate the performance of our method for population stratification correction in association analysis, we conducted a simulation study. We compared our method to a principal component analysis approach (EIGENSTRAT [24,25]) and to a model-based approach (ADMIXTURE[22]). The simulation design followed the one described in Price et al. [24] and used in Alexander et al. [22] and is outlined below. For association analysis we used a logistic regression model with additional covariates (5), which are included to correct for population structure.

logit(Y)=βX+γE (6)

Here Y is the phenotype vector, X is the genotype and E are the additional covariates. For EIGENSTRAT, we ran the analysis with 1, 2 or 10 principal components. For ADMIXTURE, we used 1 or 2 entries of their ancestry estimate. For our method we created discrete dummy variables representing community membership and plugged them into the logistic regression. We also included discrete dummy variables, which represent unconnected sub-graphs in our network. This was done in order to evaluate how well the constructed graph already represents population structure.

We considered 4 population structure scenarios suggested by the literature [24,27]:

- 2 underlying discrete subpopulations with moderate differences between cases and controls;
- 2 underlying discrete subpopulations with extreme differences between cases and controls;
- 3 underlying discrete subpopulations;
- admixed population.

For the discrete settings we simulated 1000 individuals (500 cases and 500 controls), coming from 2 or 3 populations with differentiation of Fst = 0.01. In scenario 1 60% of cases were from population 1 and 40% from population 2. In scenario 2 with more extreme mismatching cases had equal proportions between populations, whereas 100% of controls were from population 2. In scenario 3 we took the following proportions for cases and controls: 45% of cases and 35% of controls were from population 1, 35% and 20% were from population 2, 20% and 45% were from population 3. For the admixed setting we sampled individuals with ancestry proportions a and (1-a) from the 2 populations, where a is uniformly distributed. The ancestral risk was set to 3, and the probability of disease was set to 0.5 * log(*r*) * *r* ^*a*^ / (*r* − 1) as described in Price et al. [24]. For every setting we generated 100.000 random training SNPs, which were used to infer population substructure. For association testing 1 million testing SNPs in each of the 3 categories were generated:

1. Random SNPs with no disease association (same setting as for the training SNPs generation)
2. Highly differentiated SNPs with no disease association: The minor allele frequency for SNPs, coming from population 1 and 2, was set as 0.8 and 0.2 respectively.
3. Causal SNPs: Multiplicative risk model with a relative risk of 1.5.

The results are presented below in Table 7. The values are averaged across 10 independent simulation runs.We included also a 'naive' setting, which is the false positive rate (or true positive for causal SNPs) for a logistic regression without covariates. For all three discrete settings one can see that already the inclusion of the unconnected sub-graphs as covariates in a logistic regression performs similar to logistic regression with EIGENSTRAT and ADMIXTURE covariates. A further separation of the graph into communities and the inclusion them as covariates into a logistic regression improves the results in the discrete scenarios. Especially when the underlying population structure consists of three discrete populations with moderate stratification in the dataset, it can be clearly seen that our approach achieves higher power than standard approaches, while maintaining the alpha error more precisely.

**Table 7:** Average proportions of significant SNPs in the simulation study.

	naive	PCA(1 or 2 components)*	PCA (10 components)	ADMIXTURE (1 or 2 ancestry estimates)*	Unconnected components	Detected communities
**2 underlying discrete subpopulations with moderate differences between cases and controls**						
Random SNPs	0.0007397	0.0000835	0.0000879	0.0000835	0.0000841	0.0001035
Differentiated SNPs	0.8471269	0.0000849	0.0000917	0.0000833	0.0000854	0.0001003
Causal SNPs	0.5035125	0.4839071	0.4836014	0.4838919	0.4833888	0.4820485
**2 underlying discrete subpopulations with extreme differences between cases and controls**						
Random SNPs	0.0349635	0.0000852	0.0000926	0.0000851	0.0000829	0.0001029
Differentiated SNPs	1	0.0000889	0.0000979	0.0000892	0.0000772	0.000094
Causal SNPs	0.5024409	0.2571964	0.2585007	0.2573263	0.2545517	0.2562946
**3 underlying discrete subpopulations**						
Random SNPs	0.0010452	0.0000867	0.0000925	0.0000867	0.0000871	0.0001042
Differentiated SNPs	0.9981074	0.0000874	0.0000936	0.0000874	0.0000864	0.0001009
Causal SNPs	0.5007428	0.4588232	0.4592651	0.4588511	0.4587604	0.4605026
**Admixed population**						
Random SNPs	0.0006067	0.0000913	0.0000972	0.0000909	0.0006084	0.0001426
Differentiated SNPs	0.7514909	0.0000912	0.0000987	0.0000911	0.7527596	0.0130633
Causal SNPs	0.5087061	0.4445503	0.4431812	0.4445344	0.5086953	0.4694344

The values in the table represent the proportions of SNPs (averaged over 10 replications) found to be significant. The significance level was set to 0.0001. The results are present for 4 scenarios, which are described in the section: "Evaluation via simulated association studies ".

* For these methods in the scenario with 3 underlying discrete subpopulations we took 2 principal components and 2 ancestry estimates, as recommended by the authors.

We expected that our method would not provide advantages over the existing methodology in the admixed scenario, but we included this scenario for completeness.As we see from Table 7 the proposed network-based approaches perform similarly to EIGENSTRAT and ADMIXTURE for random and causal SNPs from an admixed population. Only for the special admixed scenario in which the approaches are applied to differentiated SNPs, the network-based approaches are not able to maintain the significance level. However, this simulation scenario can be considered as not realistic for application.

## Discussion

In this communication, we have proposed a new approach for population structure inference, which is based on network methodology. It is straight-forward to think of individuals in a population based study as of nodes in a big network, where the edges represent the strength of their relationship, based on a given similarity measure of their genotypes (i.e. covariance). Hence, population structure can be identified by detecting network communities. We present a heuristic method on how to generate a network structure between individuals based on their genotypic information and in a second step apply community detection algorithms in order to identify subpopulations. The method is computationally fast and flexible. To test its performance, we applied it to different subpopulations from the 1000 Genomes Project and the method was able to provide a very fine resolution of the population structure. We were not only able to separate the subpopulations within continents, but also we identified homogeneous subgroups within those subpopulations. Many of them represent individuals, which were sequenced on different platforms, whereas some of them might represent other artifacts or show small communities, which might include individuals with a stronger connection to each other.It is also important to mention that already the unconnected components of the constructed graphs show a valid separation of the individual subpopulations. Hence, applying community detection algorithms would provide a finer level of homogeneous subgroups within those unconnected components.

We further conducted a simulation study and compared the performance of our method to EIGENSTRAT and ADMIXTURE. For association testing, we used logistic regression with covariates. For every method we included the corresponding population-specific covariates: principal components for EIGENSTRAT, ancestry estimates for ADMIXTURE, and binary covariates representing the community memberships for our method.The results suggest that our method corrects for population structure more effectively when individuals come from several discrete subpopulations.

Given our findings, we have shown that network based approaches bear a great potential for subpopulation detection. The integration of network based methodology is intuitive and is suitable for large sample sizes. Due to its ability to group individuals into discrete communities without any prior information, we enable more flexible ways to analyze the data, e.g. conducting association analyses separately in the identified subsamples.The visualization of different levels of discrete communities (i.e. unconnected components or detected communities) is straightforward and the interpretation of the discrete groups is natural. Especially in the case, when little is known about the underlying structure of the data, one should use our assumption free network-based method. The high research dynamic in the field of network community detection algorithms will most likely further provide interesting applications for the identification of ancestry based genetic heterogeneity in populations. However, more research has to be done on how the information on the identified network communities can more efficiently be included in regression models.

## Supporting Information

S1 Table Contingency table for American subpopulations, rows correspond to unconnected components, columns to actual subpopulations. PUR—Puerto Rican, CLM—Colombian, MXL—Mexican.
(DOCX)

S2 Table Contingency table for African subpopulations, rows correspond to unconnected components, columns to actual subpopulations. YRI—Yoruba in Nigeria, LWK—Luhya in Kenia, ASW—African ancestry in Southwest US.
(DOCX)

S3 Table Contingency table for Asian subpopulations, rows correspond to unconnected components, columns to actual subpopulations. CHS—Southern Han Chinese, CHB—Han Chinese in Beijing, JPT—Japanese in Tokyo.
(DOCX)

S4 Table Contingency table for European subpopulations, rows correspond to unconnected components, columns to actual subpopulations. GBR—British in England and Scotland, FIN—Finnish, IBS—Iberian in Spain, CEU—Utah residents with Northern and Western European ancestry, TSI—Toscani in Italy.
(DOCX)
