# Supplementary material for: Using Network Methodology to Infer Population Substructure
Source: PLoS One. 2015 Jun 22;10(6):e0130708. doi: 10.1371/journal.pone.0130708 (PMC4476755; doi:10.1371/journal.pone.0130708)
Supplement: S1 Table — PUR—Puerto Rican, CLM—Colombian, MXL—Mexican. (DOCX) [file pone.0130708.s001.docx]

**Table S1**: Contingency table for American subpopulations, rows correspond to **unconnected components**, columns to actual subpopulations

|  | CLM | MXL | PUR |
| --- | --- | --- | --- |
| 1 | 50 | 49 | 52 |
| 2 | 0 | 0 | 3 |
| 3 | 5 | 10 | 0 |

PUR - Puerto Rican, CLM - Colombian, MXL - Mexican
