# Supplementary material for: Using Network Methodology to Infer Population Substructure
Source: PLoS One. 2015 Jun 22;10(6):e0130708. doi: 10.1371/journal.pone.0130708 (PMC4476755; doi:10.1371/journal.pone.0130708)
Supplement: S2 Table — YRI—Yoruba in Nigeria, LWK—Luhya in Kenia, ASW—African ancestry in Southwest US. (DOCX) [file pone.0130708.s002.docx]

**Table S2**: Contingency table for African subpopulations, rows correspond to **unconnected components**, columns to actual subpopulations

|  | ASW | LWK | YRI |
| --- | --- | --- | --- |
| 1 | 0 | 0 | 75 |
| 2 | 0 | 87 | 0 |
| 3 | 0 | 0 | 12 |
| 4 | 49 | 0 | 0 |
| 5 | 3 | 0 | 0 |
| 6 | 3 | 0 | 0 |

YRI - Yoruba in Nigeria, LWK - Luhya in Kenia, ASW - African ancestry in Southwest US
