# Supplementary material for: Using Network Methodology to Infer Population Substructure
Source: PLoS One. 2015 Jun 22;10(6):e0130708. doi: 10.1371/journal.pone.0130708 (PMC4476755; doi:10.1371/journal.pone.0130708)
Supplement: S3 Table — CHS—Southern Han Chinese, CHB—Han Chinese in Beijing, JPT—Japanese in Tokyo. (DOCX) [file pone.0130708.s003.docx]

**Table S3**: Contingency table for Asian subpopulations, rows correspond to **unconnected components**, columns to actual subpopulations

|  | CHB | CHS | JPT |
| --- | --- | --- | --- |
| 1 | 77 | 83 | 1 |
| 2 | 16 | 8 | 0 |
| 3 | 4 | 2 | 0 |
| 4 | 0 | 0 | 11 |
| 5 | 0 | 0 | 72 |
| 6 | 0 | 0 | 5 |

CHS - Southern Han Chinese, CHB - Han Chinese in Beijing, JPT - Japanese in Tokyo
