# Supplementary material for: Using Network Methodology to Infer Population Substructure
Source: PLoS One. 2015 Jun 22;10(6):e0130708. doi: 10.1371/journal.pone.0130708 (PMC4476755; doi:10.1371/journal.pone.0130708)
Supplement: S4 Table — GBR—British in England and Scotland, FIN—Finnish, IBS—Iberian in Spain, CEU—Utah residents with Northern and Western European ancestry, TSI—Toscani in Italy. (DOCX) [file pone.0130708.s004.docx]

**Table S4**: Contingency table for European subpopulations, rows correspond to **unconnected components**, columns to actual subpopulations

|  | CEU | FIN | GBR | IBS | TSI |
| --- | --- | --- | --- | --- | --- |
| 1 | 56 | 1 | 84 | 6 | 0 |
| 2 | 0 | 0 | 4 | 0 | 0 |
| 3 | 0 | 74 | 0 | 0 | 0 |
| 4 | 0 | 5 | 0 | 0 | 0 |
| 5 | 0 | 13 | 0 | 0 | 0 |
| 6 | 0 | 0 | 0 | 8 | 0 |
| 7 | 17 | 0 | 0 | 0 | 0 |
| 8 | 12 | 0 | 0 | 0 | 0 |
| 9 | 0 | 0 | 0 | 0 | 89 |
| 10 | 0 | 0 | 0 | 0 | 9 |

GBR - British in England and Scotland, FIN - Finnish, IBS - Iberian in Spain, CEU - Utah residents with Northern and Western European ancestry, TSI - Toscani in Italy
